# Can targeted messages reduce COVID-19 vaccination hesitancy? A randomized trial

**DOI:** 10.1016/j.pmedr.2022.101903

**Published:** 2022-07-11

**Authors:** J. Lucas Reddinger, David Levine, Gary Charness

**Affiliations:** a Department of Economics, University of California, Santa Barbara, 93106, United States; b Menard Family Initiative, College of Business Administration, University of Wisconsin-La Crosse, 54601, United States; c Haas School of Business, University of California, Berkeley, 94720, United States

**Keywords:** COVID-19, Vaccine hesitancy, Vaccination, Public health, Preventive health behavior, Behavioral public policy

## Abstract

**Background:**

Widespread vaccination is certainly a critical element in successfully fighting the COVID-19 pandemic. We apply theories of social identity to design targeted messaging to reduce vaccine hesitancy among groups with low vaccine uptake, such as African Americans and political conservatives.

**Methods:**

*Participants.* We conducted an online experiment from April 7 to 27, 2021, that oversampled Black, Latinx, conservative, and religious U.S. residents. We first solicited the vaccination status of over 10,000 individuals. Of the 4,609 individuals who reported being unvaccinated, 4,190 enrolled in our covariate-adaptive randomized trial.

*Interventions.* We provided participants messages that presented the health risks of COVID-19 to oneself and others; they also received messages about the benefits of a COVID-19 vaccine and an endorsement by a celebrity. Messages were randomly tailored to each participant’s identities—Black, Latinx, conservative, religious, or being a parent.

*Outcomes.* Respondents reported their intent to obtain the vaccine for oneself and, if a parent, for one’s child.

**Results:**

We report results for the 2,621 unvaccinated respondents who passed an incentivized manipulation check. We find no support for the hypothesis that customized messages or endorsers reduce vaccine hesitancy among our segments. A *post hoc* analysis finds evidence that a vaccine endorsement from Dr. Fauci reduces stated intent to vaccinate among conservatives.

**Conclusions:**

We find no evidence that tailoring public-health communication regarding COVID-19 vaccination for broad demographic groups would increase its effectiveness. We recommend further research on communicators and endorsers, as well as incentives.

## Highlights

• We find no evidence that tailoring public health communication regarding COVID-19 vaccination for broad demographic groups would increase its effectiveness.
• A *post hoc* analysis finds that a vaccine endorsement from Dr. Fauci reduces stated intent to vaccinate among conservatives.
• We recommend further research on communicators and endorsers, as well as incentives.

## 1 Introduction

Vaccine hesitancy has prolonged the COVID-19 pandemic in the U.S. Overcoming vaccine hesitancy is complicated because the reasons for resisting vaccination can be demographic-specific. For example, hesitancy regarding COVID-19 vaccination is higher among political conservatives and African Americans; some surveys also find increased hesitancy among Latinx people, religious Christians, and parents (Latkin et al., 2021, Milligan et al., 2021, Khubchandani and Macias, 2021, Momplaisir et al., 2021a, Momplaisir et al., 2021b, Tram et al., 2021, Riad et al., 2021a).

We apply theories of social identity to design messaging to reduce vaccine hesitancy among specific population segments. We test whether respondents report greater intent to take a hypothetical vaccine after receiving messages targeted to their demographic segment.

### 1.1 Studies on vaccine hesitancy

Dubé et al. (2015) and Aw et al. (2021) nicely summarize the literature on vaccine hesitancy. Here we discuss factors emphasized in the standard model and in theories referencing one’s sense of identity.

Prior research on health decisions often uses a rational costs-benefits framework (e.g., Strecher and Rosenstock, 1997, Armitage and Conner, 2001; on COVID-19 specifically, Kreps et al., 2020, Riad et al., 2021b). These approaches highlight:

• the seriousness of the disease,
• the safety of the vaccine,
• the effectiveness of the vaccine,
• the vaccination benefits for self and important others, and
• the expertise of the source of the message.

### 1.2 Theories of identity

In theories of identity, one learns appropriate behavior for one’s identity, typically by observing high-status individuals and the behavior of like people (Akerlof and Kranton, 2000, Stryker and Burke, 2000, Carter and Danielle, 2015). They then prefer to engage in those activities, all else equal.

One definition of social identity involves one’s sense of self, derived from perceived membership in social groups. Belonging may provide a sense of identity. Researchers have used group identity to shed light on phenomena such as ethnic and racial conflicts (Sen, 2007), discrimination, political campaigns, and human-capital formation (Coleman, 1961). Charness and Chen (2020) survey the effects of social identity on economic decisions.

Studies of vaccine hesitancy have emphasized that social and identity factors loom large (Aw et al., 2021). For example, “people tend to be more sensitive to social information that is provided to them by prestigious individuals” (Romaniuc et al., 2021). Marketing has long targeted most of the segments we study (e.g., see Podoshen, 2008, Wechsler and Wernick, 1992, Wingrove et al., 2007).

Identity can have effects on both beliefs and preferences (Charness and Chen, 2020).

In terms of belief:

• Genes generally affect one’s response to drugs. Thus, different groups (such as African Americans) may perceive evidence on vaccine efficacy as more relevant if the trials included a meaningful share of African Americans.
• People may place more trust in the benevolence of experts with greater shared identity.
• One who sees many like people engaged in an activity may decide that they have relevant information and follow the herd (as in models of information cascades, e.g., Bikhchandani et al., 1992).
• Vaccination that speeds the return to an activity a group member valued (e.g., religious services for those who had attended regularly pre-pandemic) is more important.
• People more altruistic toward those with aligned identities may be more concerned with how their own vaccination protects these people.

Identity can also affect preferences:

• One concerned about status within a group may follow the advice or actions of high-status people in the group.
• People may follow their perceptions of typical group behavior (“descriptive norms”) or of what the group considers proper behavior (“prescriptive norms”).
• If people internalize group norms, they may follow high-status leaders or their perception of common activities, as either can signal the relevant group norms.

### 1.3 Hypotheses

An individual may possess multiple identities—Black or African American, Hispanic or Latina/o/x, religiously observant (prior weekly participation), politically conservative, and an active parent. Consider non-targeted messages that promote COVID-19 vaccination and messages tailored to these specific segments of the population.

Targeted messages may heighten attention to (or the salience of) aspects of the vaccination decision of particular importance to the individual. Religious individuals may focus on the possibility of the return of church services. Black or Latinx individuals may focus on the pandemic’s disproportionate impact on their own community.

Relative to generic messages, targeted messages may also carry additional information. Individuals may learn that vaccine trials include genetic diversity. The informational content of an endorsement from someone with shared identity may be more trustworthy. An endorsement from a high-status group member may also convey group norms.

An individual who is a member of any of our five segments of interest may receive treatment of identity-targeted messages that promote COVID-19 vaccination. We hypothesize that the average marginal effect of an additional identity-concordant message has a positive effect on an individual’s intent to vaccinate. We further hypothesize that, among conservatives, an endorsement from Donald Trump is more effective than alternatives.

## 2 Methods

We conduct a randomized trial with online survey respondents. Following instructions and consent, we survey demographics, ask each respondent to read ten messages carefully to answer an incentivized question regarding message content, and finally elicit vaccination intention. The messages are randomly tailored to each respondent’s segments.

For example, a Black respondent might receive a control message with a photo of Dr. Anthony Fauci (who is white), or a targeted message with a photo of COVID-19 vaccine co-developer Kizzy Corbett (who is Black). The text might refer to the average risk of COVID-19, or it might emphasize that African Americans are more likely to suffer from COVID-19.

### 2.1 Messages

Our baseline messages emphasize the health risks of COVID-19 and the safety and benefits of a hypothetical COVID-19 vaccine (Table 1). We randomized message components for specific segments (Appendix A). For respondents eligible for more than one message, we randomized the several message components with equal probability, balanced on segments. Nearly all messages were accompanied by photos. Importantly, either all possible treatments for a given component had corresponding photos or none did.

**Table 1:** Baseline messages.

Element name	Text
*(all received)*	Consider a COVID-19 vaccine described by the following:
Population tested	The vaccine has been approved by a rigorous FDA process involving tens of thousands of people.
Trial results	This randomized trial found very high effectiveness and almost no serious side effects.
Impact	COVID-19 has infected over 30 million Americans, leading to over 500,000 deaths.
Protection	When you get vaccinated, you help protect yourself and the people around you from this virus.
Elders	We must protect our elders and get vaccinated! *(Photo of an elder and a child.)*
Gatherings	You can make up for missed get-togethers with friends and family once everyone has been vaccinated. *(Photo of a wedding.)*
Availability	The vaccine is available at your doctor’s office and local pharmacies.

*Note:* See Appendix Table 5 for all treatment messages.

#### 2.1.1 Danger of COVID-19

All respondents read, “COVID-19 has infected over 30 million Americans, leading to over 500,000 deaths.” A random subset of Black and Latinx respondents also read about the higher impact on their community. A separate randomization of the religiously observant read that the virus has spread frequently in their place of worship (church, synagogue, mosque, or temple, each with an appropriate photo).

#### 2.1.2 Vaccine safety

**Fig. 1:**
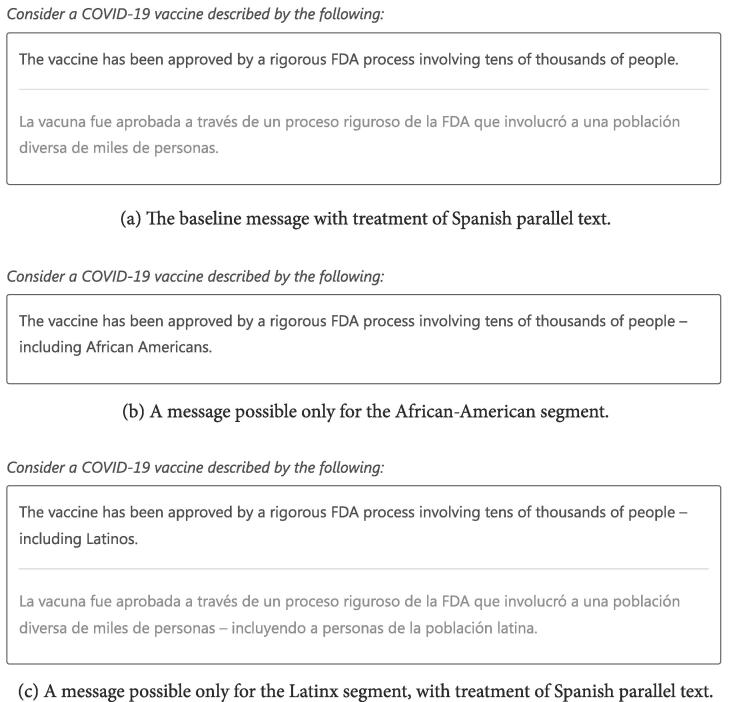
Example messages on an FDA trial.

All respondents read, “The vaccine has been approved by a rigorous FDA process involving tens of thousands of people. This randomized trial found very high effectiveness and almost no serious side effects.” African Americans and Latinx people were randomized to also read that the trial included people from their group. Fig. 1 depicts examples.

#### 2.1.3 Parenting

Parents randomly received, “Children are at risk of long-term damage to their lungs and other organs. Nobody is sure how common or long-lasting this damage will be.” A photo of children was included; Latinx parents saw children in a Hispanic parade.

#### 2.1.4 Spillovers to the community

Infectious diseases have large negative externalities in communities. Thus, concern for others can be a major predictor of willingness to vaccinate. Everyone received, “The elderly are most at risk for COVID-19. Unfortunately, some cannot be vaccinated because of health conditions.”

This was followed with a randomized control message, “We must protect our elders and get vaccinated!” Parents randomly received this instead: “Imagine what you would feel like if you did not vaccinate your child, and then an elderly person in your home became ill.” This included a photo of two grandparents playing with grandchildren. Conservatives randomly received this instead: “We share small-town values like caring for our neighbors—especially elders.” Finally, a subset of religious respondents read this: “The Bible tells [Our holy books tell] us to care for those most vulnerable.”

Finally, everyone received the message, “When you get vaccinated, you help protect yourself and the people around you from this virus.”

#### 2.1.5 Benefits: Ending social isolation

**Fig. 2:**
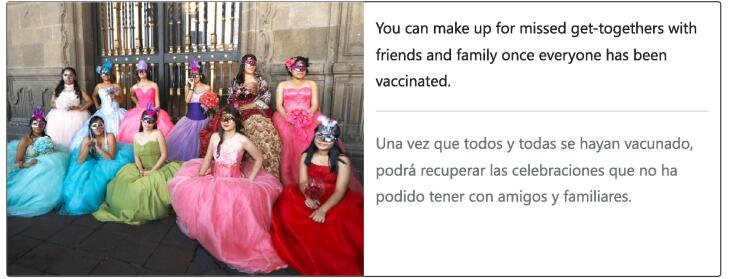
A message on gatherings with treatment for the Latinx segment and with treatment of Spanish parallel text. Photo credit: la Secretaría de Cultura de la Ciudad de México.

Our control condition explains, “You can make up for missed get-togethers with friends and family once everyone has been vaccinated.” Latinx respondents randomly received an accompanying photo of a *quinceañera*, celebrating the fifteenth birthday of a young Latina (see Fig. 2). Parents randomly received, “You can make up for missed children’s parties and outings with friends and family once everyone has been vaccinated,” alongside a photo of a children’s party. Religious respondents randomly received, “You can safely attend [place of worship] with friends and family once everyone has been vaccinated,” with a photo of the respective place of worship. All respondents then received, “These events will be so much nicer when they are safe.”

In an additional randomization, some religious conservatives received, “Freedom to go to church is the freedom to worship together, not infect each other!”

#### 2.1.6 Availability

Respondents received a control message: “The vaccine is available at your doctor’s office and local pharmacies.”

Some religious and parents also read that the vaccine is available at their place of worship or their child’s school. These locations increase convenience, imply an endorsement by their religious group or school, and suggest that vaccines are normative for that group.

#### 2.1.7 Language

Latinx respondents were randomly treated with Spanish parallel text for all messages received.

#### 2.1.8 Other messages components

We additionally randomized the following treatments:

• Conservatives randomly received, “When you get vaccinated, you help protect your body and your mind from this nasty and foreign virus.”
• Non-Black, non-Latinx conservatives randomly received, “Republican governors from Georgia to Ohio have stressed the economic and human cost of this pandemic.”

#### 2.1.9 Recommendations

**Fig. 3:**
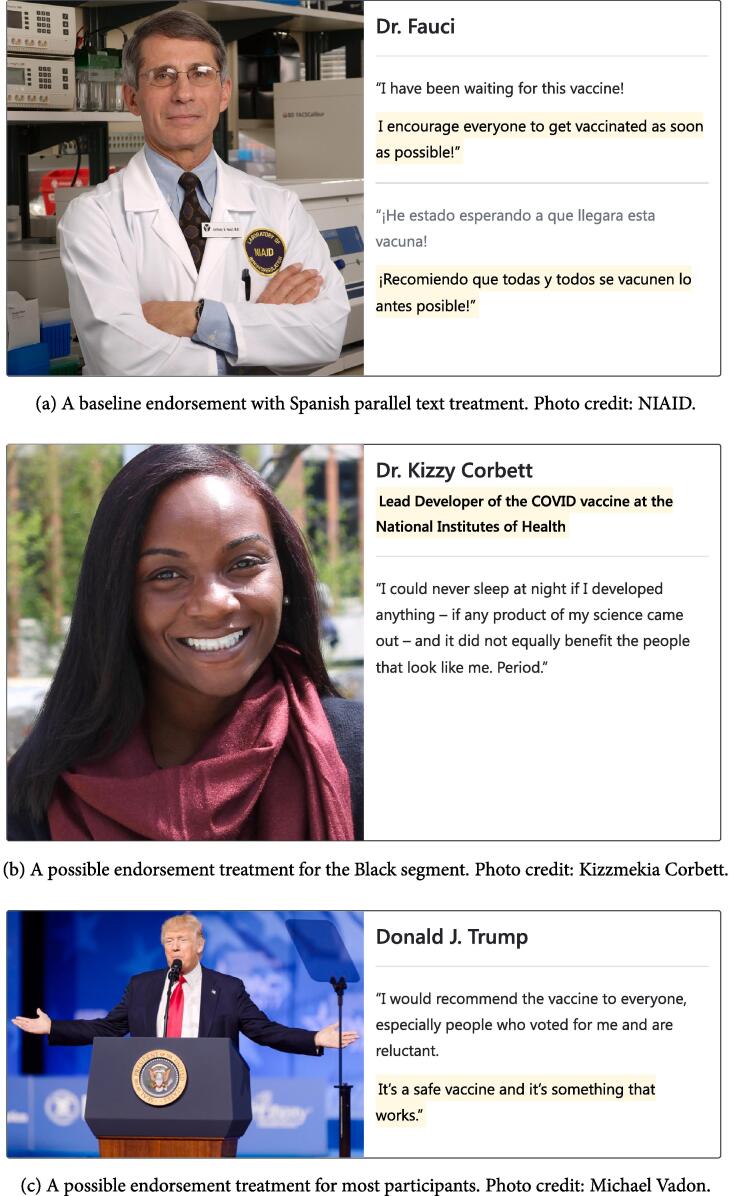
Example endorsements.

Each respondent received an endorsement by a famous person such as Dr. Fauci, Donald Trump, Barack and Michelle Obama, a famous religious leader (e.g., the Pope), or a famous entertainer or athlete (e.g., Tom Hanks, LeBron James). Fig. 3 depicts example recommendations. Some endorsers were selected to be concordant on conservatism (e.g., Trump vs. the Obamas), identifying as Latinx (e.g., Hanks vs. Jennifer Lopez), identifying as Black, or religious affiliation (Appendix Table 6, Table 7). We chose our recommenders from lists of celebrities from each segment, identifying those with a large social media presence or those recommended by consultants or pilot-survey respondents.

We gave each participant a set of messages they might receive, based on their personal characteristics. We then randomized messages. The risk sets for all respondents included a recommendation by Dr. Fauci. We included Trump and the Obamas if not a Black conservative, and Dwayne Johnson if not Latinx and age 65 or older.

The risk set for all religious respondents included a religious leader: If Black, the Reverend Warnock, a famous Black pastor and current U.S. senator. For others the endorsement came from the Pope (if Catholic or Latinx) or Rick Warren (if non-Black, non-Latinx, non-Catholic), founder of the Saddleback evangelical megachurch.

If Black, the risk sets included LeBron James or Kizzy Corbett.

If Latinx, the risk set included Alejandro Fernández (for ages over 65), Jennifer Lopez (if religious and under 65), or Bad Bunny (if non-religious and under 65). If Latinx and over 65, the risk set also included Tom Hanks and the Pope.

If neither Black nor Latinx but conservative, the set included Tom Brady. If not both religious and conservative, Tom Hanks. If neither conservative nor religious, LeBron James.

#### 2.1.10 Pre-testing messages

We qualitatively tested messages with experts on each segment. We addressed both comprehension and suitability. We then conducted a quantitative pilot where respondents rated different messages.

### 2.2 The sample

We recruited United States residents through Prolific, which maintains a participant pool for web-based research and facilitates sampling stratified on participant characteristics.

We over-sampled individuals who had told Prolific they (1) identify as Black or African American, (2) identify as Latina/o/x or Hispanic, (3) either voted for Trump in 2020 or self-reported being “conservative” on a political spectrum, or (4) reported at least weekly participation in religious activities pre-pandemic. The screening questions are in Appendix E.

In April 2021, we invited participants who met our selection criteria to take an initial single-question screening survey: “Have you already taken a COVID-19 vaccine dose?” Appendix Table 8 describes respondent demographics.

We restrict our analysis to those without any COVID-19 vaccination who correctly answered an incentivized attention check. At that time, roughly half of American adults had received at least one vaccine dose. Appendix B contains details and a sampling pipeline diagram. We stopped recruiting for the study once enrollment plateaued (Fig. 6).

Appendix F describes consent, instructions, the manipulation check, and debriefing.^1^

### 2.3 Outcome measures

Our primary outcome is the reply to: “How likely are you to take the COVID-19 vaccine described above?” Possible responses ranged from “highly unlikely” (coded as 1) to “highly likely” (7). We drop respondents who chose “Don’t know/ prefer not to say” (N=50, 1%). Parents also answered a similar question about vaccinating their child.

### 2.4 Statistical methods

We had intended to enroll 6,500 to 7,000 participants (at least 1,000 per segment). Similar studies (c.f., Freeman et al., 2021, Kreps et al., 2020) have found effects with comparable sample sizes. We were ultimately constrained by the relatively small size of the Prolific participant pool. Attrition during the sampling procedure was minimal (Appendix B).

We implement covariate-adaptive stratified block randomization given our five segments of interest, obtaining 32 strata (“subsegments”). Participants are at risk for multiple randomized treatment components given their subsegment membership. Each possible treatment is assigned with equal probability by Qualtrics survey software, maintaining balance.

Our main test examines willingness to be vaccinated depending on the number of concordant messages.^2^ We include separate intercepts for each subsegment, controlling for the respondent’s maximal possible intensity of treatment. Student’s *t*-test is then an exact test with the inclusion of subsegment fixed effects (Bugni et al., 2018). We drop subsegments with fewer than ten respondents (six subsegments, N=17).

We next estimate which message components matter. To reduce the number of tests, we consider bundles of message components—“Population tested in the trials,” “Community impact,” “Children affected,” “Protecting the elderly,” “Protection,” “Elders,” “Gatherings,” and “Availability.”

We test the joint effect of all concordant messages received by each segment: Black or African American, Latinx or Hispanic, conservative, religious, and parents.

Last, our analysis plan pre-specified a test of whether Trump is a particularly effective endorser among conservative respondents.

## 3 Results

### 3.1 Descriptive analysis

**Fig. 4:**
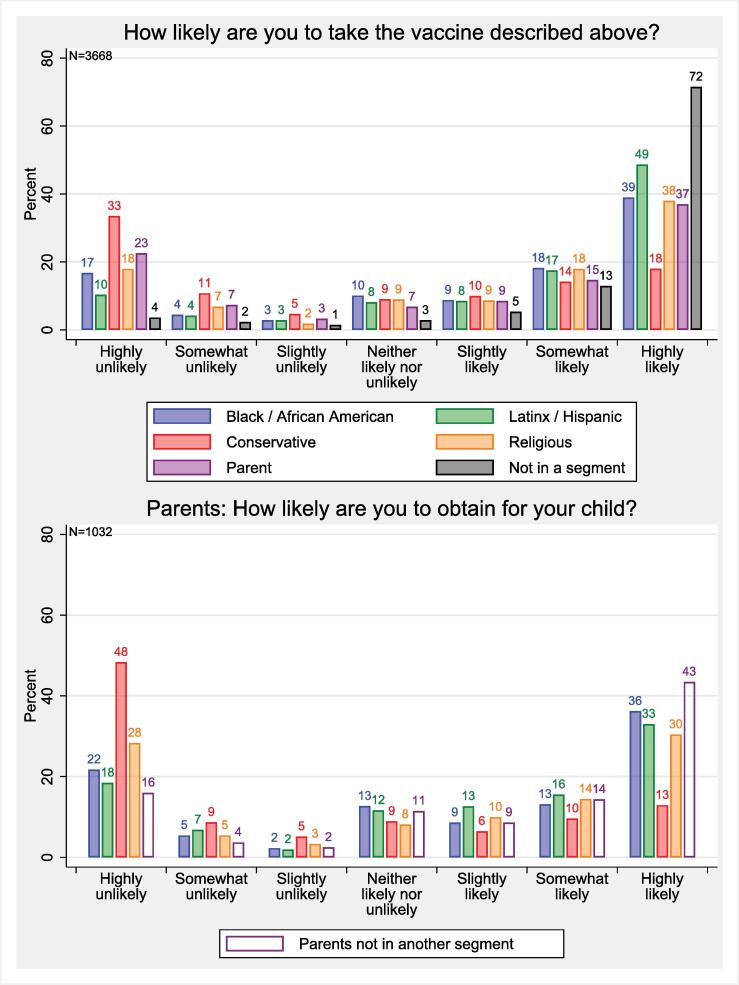
Distribution of likelihood to accept the vaccine described.

Table 2 displays summary statistics: 46% were “highly likely” and 15% were “highly unlikely” to get vaccinated, with other replies scattered (Fig. 4). Intention-to-vaccinate children (mean 4.16, range 1 to 7) was lower than intention-to-vaccinate self (4.63).

**Table 2:** Summary statistics.

	Intent to vaccinate self				Intent to vaccinate child*			
	N	Mean	Prob. equals	% Highly	N	Mean	Prob. equals	% Highly
		intent	no segments	unlikely		intent	no segments	unlikely
Black	675	5.00	0.00	17%	221	4.65	0.04	22%
		(2.25)				(2.38)		
Latinx	602	5.47	0.00	10%	103	4.72	0.15	18%
		(2.03)				(2.28)		
Conservative	1174	3.66	0.00	33%	449	2.97	0.00	48%
		(2.38)				(2.30)		
Religious	719	4.89	0.00	18%	332	4.31	0.00	28%
		(2.31)				(2.48)		
Parent	1093	4.63	0.00	23%				
		(2.44)						

Overall	3668	5.18		15%	1032	4.16		30%
		(2.26)				(2.48)		
A member of	2638	4.75	0.00	20%	788	3.87	0.00	34%
⩾1 segment		(2.36)				(2.47)		
A member of	1030	6.29		4%	244	5.10		16%
no segments		(1.48)				(2.23)		

*Notes:* Standard deviations in parentheses. Intent of 1 corresponds to “highly unlikely” to vaccinate, while 7 is “highly likely.” We intentionally over-sampled our demographics of interest, so our sample is not representative, and the means above are unweighted. Many respondents are in more than one segment (e.g., Latinx and Religious and Parent). Because respondents in different segments received different combinations of message elements, the means are not directly comparable. This table uses the sample for descriptive statistics (see Appendix Fig. 7 for the sampling flowchart).

* For intent to vaccinate child, the sample is restricted to parents; accordingly “a member of ⩾1 segment” considers only the non-parent segments, as does “a member of no segments.”

Latinx individuals were relatively high (5.47), but Black individuals (5.00), the religious (4.89), and parents (4.63) showed lower willingness. Conservatives were the negative outlier (3.66). A full third (33%) of conservatives reported they were “highly unlikely” to accept the vaccine, more than twice the average. Those not in any segment had mean intention-to-vaccinate of 6.29, higher than the focal segments.

### 3.2 Do concordant messages increase likelihood to vaccinate?

**Fig. 5:**
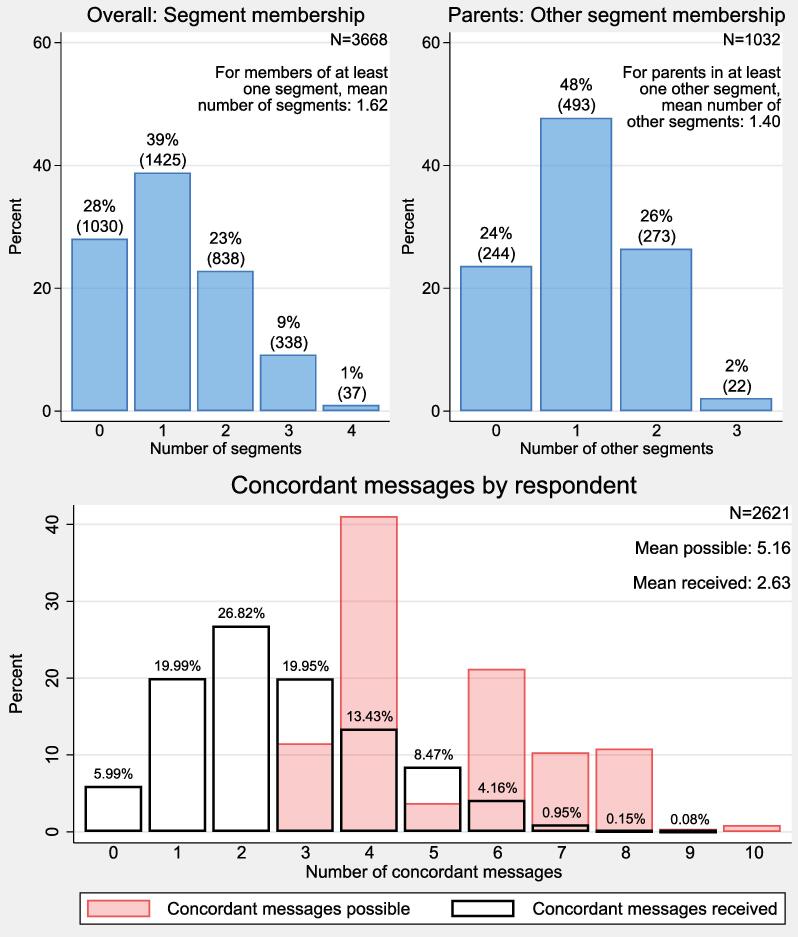
Sample characteristics: segment membership and condordant messages.

**Fig. 6:**
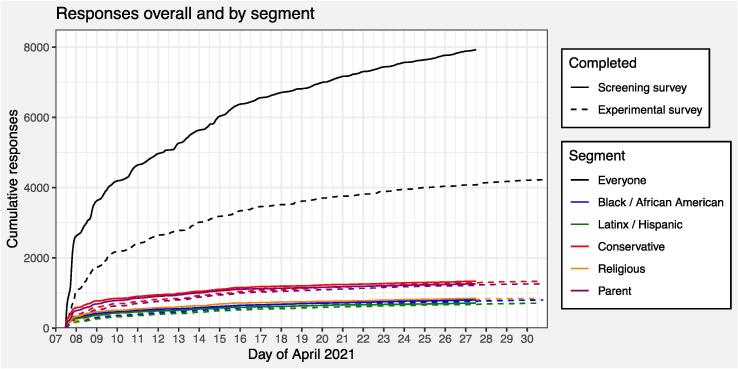
Cumulative responses over time by segment.

Our sample for the experiment included 2,621 respondents who were members of at least one segment (mean membership of 1.62 segments). The mean number of identity-tailored messages possible for a participant was 5.16. Fig. 5 shows histograms of treatment intensity; Appendix C offers additional tabulations.

Table 3 contains our primary results. Our analysis uses ordered-logit specifications (similar results using ordinary least squares available upon request). We find no evidence of a relationship between the number of concordant messages received and reporting a greater intention-to-vaccinate. Results for parents’ intention-to-vaccinate child are similar in having a positive sign, a small magnitude, and lack of statistical significance.

**Table 3:** Does receipt of concordant messages increase willingness to vaccinate?

Panel A. Effect of concordant score on intent to vaccinate								
	Ordered logit							
	Intent to vaccinate self	Intent to vaccinate child						
Concordant score	0.018	0.032						
	[-0.041,-0.076]	[-0.055,-0.120]						
Cut 1	−2.399***	−1.710***						
	[-2.694,-2.103]	[-2.018,-1.402]						
Cut 2	−1.959***	−1.389***						
	[-2.248,-1.671]	[-1.691,-1.086]						
Cut 3	−1.782***	−1.219***						
	[-2.068,-1.496]	[-1.517,-0.920]						
Cut 4	−1.336***	−0.688***						
	[-1.618,-1.055]	[-0.980,-0.397]						
Cut 5	−0.880***	−0.289*						
	[-1.158,-0.601]	[-0.577,-0.002]						
Cut 6	−0.103	0.384**						
	[-0.377,-0.172]	[-0.097,-0.672]						
Subsegments	24	11						
Observations	2621	1032						
*Notes:* 95% confidence intervals in brackets using heteroskedasticity-robust standard errors. Each regression includes subsegment fixed effects. Outcome ranges from 1 (highly unlikely) to 7 (highly likely). Concordant score is the number of message attributes customized for that respondent’s segment memberships, plus an additional unit if treated with Spanish parallel text if Latinx. ^+^p<0.10, * p<0.05, ** p<0.01, *** p<0.001.								
								
Panel B. Margins of coefficient on concordant score								

		Δ Prob. of each reply: 1 is “highly unlikely to vaccinate,” 7 is “highly likely”						
	N	1	2	3	4	5	6	7

Self	2621	−0.0026	−0.0005	−0.0002	−0.0003	−0.0002	0.0002	0.0035
Child	1032	−0.0058	−0.0005	−0.0002	−0.0003	0.0001	0.0008	0.0057

*Notes:* The marginal change in the likeliness of reporting the given category of vaccination intent due to an increase of one concordant element, based on the ordinal logit estimates in Panel A.

We then tested which message components matter: if the vaccine was tested on a population including one’s own group (pooling Black and Latinx segments); if the gatherings enabled by the vaccine are highly relevant to your group (pooling Latinx, conservative, religious and parent segments); “Impact” messages (including Church impacts); “Elders” messages; “Protection” messages; “Gatherings” messages; and “Availability” messages (Appendix Table 11). Consistent with Table 3, the coefficients are collectively not statistically significant ($\chi_{8}^{2}=5.39,\;p=0.715$).

We next tested if concordant messages might matter for a specific segment (Appendix Table 12). There is no evidence that having concordant messages is statistically significantly useful for any of our five segments ($\chi_{5}^{2}=1.54,\;p=0.908$).

### 3.3 Does Trump matter specifically for conservatives?

Conservatives are the most vaccine-hesitant group. We pre-specified one celebrity endorsement as most important—the effect of Trump, who at times recommended vaccination. To reduce the number of subsegments and comparison recommenders, we focus on non-Black, non-Latinx conservatives. Results, with Trump as the baseline recommender, are shown in Table 4.

**Table 4:** Comparison of recommendations among conservatives.

	Ordered logit (Reference recommender: Donald Trump)	
	Intent to vaccinate self	Intent to vaccinate child
The Obamas	−0.003	0.281
	[-0.392,-0.387]	[-0.423,0.985]
Dr. Fauci	−0.618**	−0.136
	[-1.012,-0.223]	[-0.847,0.576]
Dwayne “The Rock” Johnson	−0.305	−0.107
	[-0.695,-0.086]	[-0.890,0.676]
Tom Brady	−0.044	0.563
	[-0.449,-0.362]	[-0.161,1.288]
Tom Hanks	−0.332	0.241
	[-0.752,-0.089]	[-0.618,1.100]
The Pope^†^	−1.104*	
	[-1.969,-0.239]	
Rick Warren	−0.208	0.285
	[-0.786,-0.369]	[-0.577,1.147]
ℙ(allotherrecommenders=Trump)	0.007**	0.347
Recommender risk-sets^‡^	4	2
Observations	963	381

*Notes:* 95% confidence intervals in brackets using heteroskedasticity-robust standard errors. Outcome ranges from 1 (highly unlikely) to 7 (highly likely). Statistical tests comparing all pairs of recommenders are in Appendix Table 13. All risk sets included recommendations from Trump, Fauci, the Obamas, Johnson, and Brady. Religious Catholics also included the Pope, other religious included Warren, and non-religious included Hanks.

† Recommenders and recommender risk sets with fewer than three observations dropped.

‡ Regressions include recommender risk-set fixed effects.

^+^p<0.10, * p<0.05, ** p<0.01, *** p<0.001.

Conservatives are almost equally responsive to the Obamas (β=-0.003, 95% CI =[-0.392,0.387],p=0.99) and not detectably less responsive to Tom Brady (a prominent conservative, β=-0.044, 95% CI =[-0.449,0.362],p=0.833), both relative to Trump.

The other possible recommenders were slightly less effective than Trump. The joint test shows Trump is distinct on average from the seven alternatives (for Trump versus all others, $\chi_{7}^{2}=19.45,\;p=0.007$). At the same time, only the coefficient on Fauci is significantly different from the effect of a Trump recommendation (β=-0.618, 95% CI =[-1.012,0.223],p=0.002). Note that this last Fauci test was not pre-registered.

In short, the results support the hypothesis of Trump’s effectiveness with conservatives. Equally, Tom Brady and the Obamas appear roughly as effective as Trump, even among conservatives.

## 4 Discussion

### 4.1 Summary

We surveyed 3,668 unvaccinated Americans in April 2021 about their likelihood of getting vaccinated, using messages with specific characteristics and celebrity endorsements. Our experiment involved 2,621 participants who were members of at least one of five important demographic segments—Black, Latinx, conservative, religious, and parents—when about half of American adults were unvaccinated.

As others have found, vaccine hesitancy is above average for Black and Latinx respondents and much higher for conservatives.

Contrary to our hypotheses, receiving more concordant messages regarding the vaccine had no detectable effect on stated willingness to vaccinate. Our sample size was large enough to detect effects (c.f., Freeman et al., 2021, Kreps et al., 2020) and Prolific is a well-respected subject pool. While our negative results could reflect methodological issues (limitations listed below), our results suggest any effects are modest at best.

In exploratory tests, no segment had a large benefit from concordant messages. Furthermore, no message element (such as dangers of COVID-19 segment-customized or having a recommender from the same segment) had a large effect.

With caution regarding multiple-hypothesis testing, we find mixed evidence that Trump is a particularly effective recommender for conservatives, and a hint that Dr. Fauci is especially unconvincing for conservatives.

### 4.2 Implications

Despite our findings, it remains sensible to customize messages for segments.

In October 2021, Larsen et al. (2022) treated U.S. counties with a large-scale advertising campaign featuring a COVID-19 vaccine endorsement by Donald Trump on Fox News, finding evidence of increased vaccination at average cost of about $1 per vaccination. Other studies have also found Trump promoting the vaccine has a positive effect on intent (Kreps et al., 2020, Bokemper et al., 2021). While our evidence weakly supports the effectiveness of a Trump endorsement, it is not clearly more effective than all alternatives.

We attribute this discrepancy to the timing of the studies and the impact of the message. Bokemper et al., 2021, Kreps et al., 2020 found Trump endorsement effective for a hypothetical vaccine during Summer 2020, months before the first emergency use authorization. We sampled unvaccinated respondents in April 2021, when half of U.S. adults had been vaccinated. Our sample was thus more vaccine-hesitant than these other studies by construction. Further, political discourse had galvanized beliefs and attitudes regarding vaccination, reducing the possible effect of our study. The success of the Larsen et al. (2022) trial is likely due to their video’s effectiveness, in addition to their larger sample size.^3^,^4^

If public-service messages like ours cannot overcome most vaccine hesitancy, more costly interventions may nevertheless be cost-effective. For example, perhaps personal communication from friends and family or from a family doctor is more important than marketing messages.

Moving beyond traditional social-marketing approaches, evidence generally supports the effectiveness of monetary incentives and lotteries (Campos-Mercade et al., 2021, Barber and West, 2022). Tying privileges, such as school enrollment or riding commercial airlines, to vaccination status may also motivate some people (Oliu-Barton et al., 2022, Mills and Rüttenauer., 2022).

We finally consider implications for theories of identity, which are supported by both many published studies and introspection. We worry that publication bias may lead to under-reporting of other negative findings.^5^ Theories of identity are not always easy to exploit. We need much more research to explore the boundary conditions.

### 4.3 Limitations

The survey only reported on willingness to vaccinate, not vaccination.

In addition, the pool of Prolific respondents was not necessarily representative of their segments. Still, this is not a concern unless the resulting bias is correlated with treatment.

We defined membership in our “conservative” segment as either Trump voters or self-identified conservatives. Some Trump voters are not conservative, and vice versa.

Furthermore, our findings do not reflect the effects of any targeted messaging prior to our trial, since we collected data after half of American adults had already received at least one vaccine dose.

Finally, it is important to test additional message elements, more realistic messaging, more messengers, and in different regions.

## Funding statement

The Center on the Economics and Demography of Aging (NIH 2P30AG012839), University of California, Berkeley, provided funding.

## CRediT authorship contribution statement

**J. Lucas Reddinger:** Conceptualization, Methodology, Formal analysis, Project administration, Software, Validation, Investigation, Resources, Data curation, Visualization. **David Levine:** Conceptualization, Methodology, Formal analysis, Project administration, Funding acquisition. **Gary Charness:** Conceptualization, Methodology, Formal analysis, Project administration.

## Declaration of competing interest

The authors declare that they have no known competing financial interests or personal relationships that could have appeared to influence the work reported in this paper.

## Figures and Tables

**Table 5:** Summary of possible messages by segment.

Element name	Segments	Variations on text
Population tested	*All*	The vaccine has been approved by a rigorous FDA process involving tens of thousands of people.
	Black or Latinx	The vaccine has been approved by a rigorous FDA process involving tens of thousands of people—including African Americans [Latinos].
Trial results	Baseline	This randomized trial found very high effectiveness and almost no serious side effects.
Impact	Baseline	COVID-19 has infected over 30 million Americans, leading to over 500,000 deaths.
	Black or Latinx	*Adds:* The African American [Latino/a/x] community has been especially hard-hit by this virus.
	Conservative	*Adds:* Republican governors from Georgia to Ohio have stressed the economic and human cost of this pandemic.
*Impact – Churches*	Religious	The virus has spread frequently in churches [synagogues / mosques / temples]. *(Photo of matching religious institution.)*
*Children*	Parent	Children are at risk of long-term damage to their lungs and other organs. Nobody is sure how common or long-lasting this damage will be. *(Photo of children.)*
	Parent and Latinx	*Instead uses a photo of children in a Hispanic parade.*
Elders	*All*	We must protect our elders and get vaccinated! *(Photo of an elder and a child.)*
	Parent	Imagine what you would feel like if you did not vaccinate your child, and then an elderly person in your home became ill. *(Photo of grandparents playing with grandchildren.)*
	Conservatives	We share small-town values like caring for our neighbors—especially elders. *(Photo of an elder and a child.)*
	Religious	The Bible tells [Our Holy Books tell] us to care for those most vulnerable. *(Photo of a Bible or a generic Holy Book.)*
Protection	Baseline	When you get vaccinated, you help protect yourself and the people around you from this virus.
	Conservatives	When you get vaccinated, you help protect your body and your mind from this nasty and foreign virus.
Gatherings	Baseline	You can make up for missed get-togethers with friends and family once everyone has been vaccinated. *(Photo of a wedding.)*
	Latinx	*Instead uses a photo of a quinceañera, a coming-of-age party for a young Latina.*
	Parent	You can make up for missed children’s parties and outings with friends and family once everyone has been vaccinated. *(Photo of a child’s birthday party.)*
	Religious	You can safely attend church [synagogue / mosque / temple] with friends and family once everyone has been vaccinated.
*Gatherings – Freedom*	Christian and conservative	Freedom to go to church is the freedom to worship together, not infect each other!
Gathering safety	*All*	These events will be so much nicer when they are safe.
Availability	Baseline	The vaccine is available at your doctor’s office and local pharmacies.
	Parent	*Adds:* …and your child’s school.
	Religious	*Adds:* …and your church [synagogue / mosque / temple].
Recommendation	*All*	*See*Table 6, Table 7*.*
*Spanish language*	Latinx	*Text of all messages also presented in Spanish, below the English.*

*Notes:* If there is a “baseline” row for an element, then everyone received a message for that element. If there is no baseline message (those with an italicized name in the first column), then half of each eligible segment received a message, and half received no message for that element. If a respondent matched with more than one segment and message for a given element, then they were randomized with equal probability for all eligible messages. “Churches” changed to temples for Buddhists or Mormons, to synagogues for Jews, and to mosques for Muslims. “The Bible” changed to “Holy Books” if religious and not Christian or Jewish. Only those who report practicing at least weekly are at risk of religious messages.

**Table 6:** Endorsers.

Endorser	Endorsement shown	Endorser notability
Dr. Anthony Fauci*	I have been waiting for this vaccine! I encourage everyone to get vaccinated as soon as possible!*	“Director of the U.S. National Institute of Allergy and Infectious Diseases (NIAID) and the chief medical advisor to the president…. *The New York Times* described Fauci as one of the most trusted medical figures in the United States.”
Dwayne “The Rock” Johnson*	I wish I had access to this vaccine before I was exposed to COVID. I encourage everyone to get vaccinated as soon as possible!*	“One of the greatest professional wrestlers of all time …His films have grossed over …$10.5 billion worldwide, making him one of the world’s …highest-paid actors.”
Donald Trump	I would recommend the vaccine to everyone, especially people who voted for me and are reluctant. It’s a safe vaccine and it’s something that works.	Former U.S. president
Barack and Michelle Obama	The COVID vaccine is our best shot at beating this virus, looking out for one another, and getting back to some of the things we miss. Getting vaccinated will save lives—and that life could be yours.	Former U.S. president and first lady
Tom Hanks*	I wish I had access to this vaccine before I was exposed to COVID. I encourage everyone to get vaccinated as soon as possible!*	“One of the most popular and recognizable film stars worldwide …Hanks’s films have grossed more than $9.96 billion worldwide.”
Tom Brady*	Get vaccinated so we can get our next season back to normal!*	“Brady is widely considered to be the greatest [American football] quarterback of all time.”
LeBron James*	Get vaccinated for our community. It is safe and will save lives!*	“Widely considered one of the greatest players in [National Basketball Association] history …selected to the All-NBA Team a record 13 times”
Kizzmekia “Kizzy” Shanta Corbett	I could never sleep at night if I developed anything—if any product of my science came out—and it did not equally benefit the people that look like me. Period.	“Scientific lead of the [NIH Vaccine Research Center] Coronavirus Team …propelling …a COVID-19 vaccine”
Bad Bunny*	I wish I had access to this vaccine before I was exposed to COVID. I encourage everyone to get vaccinated as soon as possible!*	“The first Latin urban music artist on the cover of *Rolling Stone* magazine …*Time* magazine named him one of the 100 most influential people in the world on their annual list (2020).”
Pastor Rick Warren*	My flock works to protect our spirits and our bodies. This vaccine is essential for protecting our bodies.*	“Founder and senior pastor of Saddleback Church, …the largest church in California …Named by *Time* as one of the ‘100 Most Influential People in the World.’ …His books have sold over 30 million copies.”
Pope Francis	I believe that morally everyone must take the vaccine. It is the moral choice because it is about your life but also the lives of others.	The Pope
Reverend Raphael Warnock	This pandemic isn’t over yet and we must all stay vigilant to protect our community. Follow public health guidance, stay distanced and get the vaccine when you are eligible.	“Senior pastor of the Ebenezer Baptist Church …Martin Luther King Jr.’s former congregation …United States senator from Georgia since 2021”
Jennifer Lopez*	I have been waiting for this vaccine! I encourage everyone to get vaccinated as soon as possible!*	“One of the highest-paid Latin actresses worldwide …a pop culture icon”
Alejandro Fernández*	I have been waiting for this vaccine! I encourage everyone to get vaccinated as soon as possible!*	“Sold over 20 million records worldwide, making him one of the best-selling Latin music artists.”

*Notes:* Sorted by risk (descending). All endorser notability quotations come from the endorser’s Wikipedia page, accessed on 6 April 2021 and 16 March 2022.

* These quotes are fictitious. Others are actual exact quotes or nearly exact.

**Table 7:** Risk set for the recommendation messages.

Selection criteria							Set of recommenders, each with equal probability				
Black	Latinx	Not Black, Not Latinx	Conservative	Religious	Age ⩾65	Catholic	Fauci	Johnson	The Obamas	Trump	Others
Yes			No	No			Yes	Yes	Yes	Yes	James, Corbett
Yes			No	Yes			Yes	Yes	Yes	Yes	James, Corbett, Warnock
Yes			Yes				Yes	Yes			James, Corbett
	Yes				Yes		Yes		Yes	Yes	Hanks, The Pope, Fernández
	Yes			Yes	No		Yes	Yes	Yes	Yes	The Pope, Lopez
	Yes			No	No		Yes	Yes	Yes	Yes	Bad Bunny
		Yes	Yes	Yes		Yes	Yes	Yes	Yes	Yes	The Pope, Brady
		Yes	Yes	Yes		No	Yes	Yes	Yes	Yes	Brady, Warren
		Yes	Yes	No			Yes	Yes	Yes	Yes	Hanks, Brady
		Yes	No	Yes		Yes	Yes	Yes	Yes	Yes	Hanks, The Pope
		Yes	No	Yes		No	Yes	Yes	Yes	Yes	Hanks, Warren
		Yes	No	No			Yes	Yes	Yes	Yes	Hanks, James

**Table 8:** Participant sampling and recruitment

		Screening survey*			Experimental survey^**^				
Segment	Potential subjects	Total	Unvaccinated		Invited	Completed		Unvaccinated	
Overall	44800	7932	4609	58%	4609	4225	92%	4072	96%
Black	3200	1599	916	57%	916	817	89%	784	96%
Latinx	3300	1101	595	54%	595	523	88%	500	96%
Conservative	2400	1321	899	68%	899	832	93%	816	98%
Religious	9000	2032	783	39%	783	687	88%	662	97%
Unvaccinated	4300	1978	1519	77%	1519	1467	97%	1410	96%

*Notes:* These demographic characteristics were volunteered to Prolific by the participants prior to our survey experiment; accordingly, these characteristics are underreported. All other tables use demographic characteristics reported in our survey. We recruited only U.S. residents with a 98% approval rate on Prolific.

* Excludes any participant who shares an IP (Internet Protocol) address with another participant.

** Includes all participants who reported vaccination status, regardless of the number of surveys attempted, manipulation check, or vaccination intent.

**Fig. 7:**
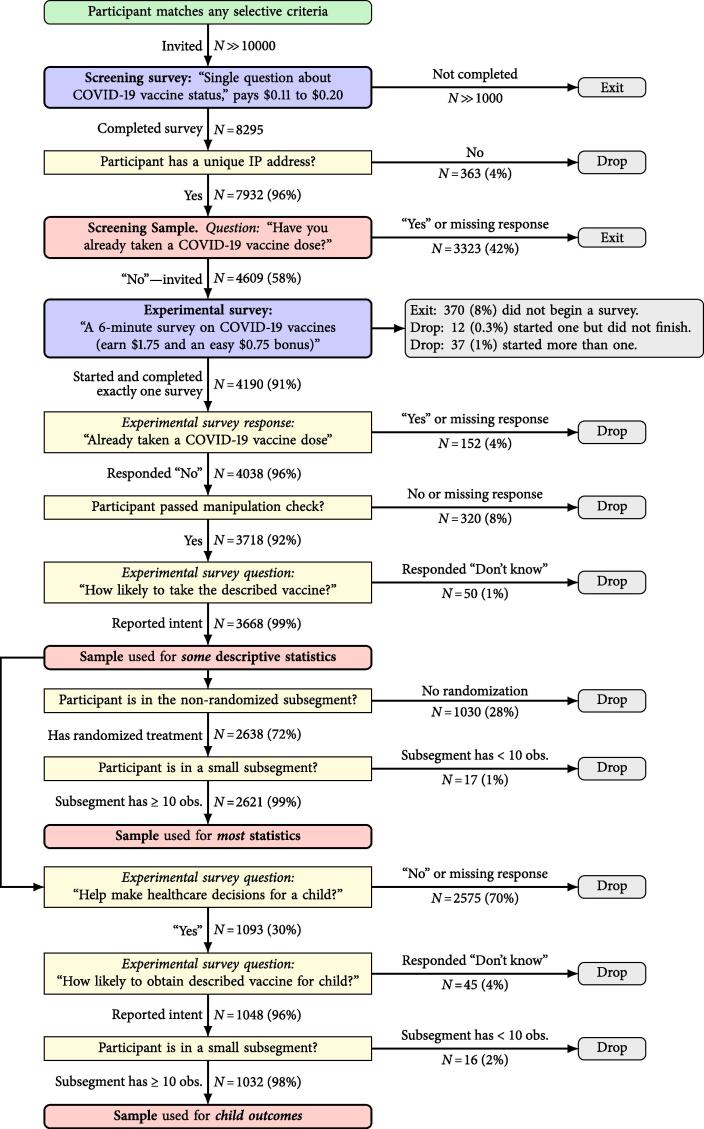
Participant sampling flow chart.

**Table 9:** Participants by segment.

Segment	N	Percent
Black / African American	675	18%
Latinx	602	16%
Conservative	1174	32%
Religious	719	20%
Parent	1093	30%
A member of no segments	1030	28%

Total	3668	

*Notes*: We collected these demographic characteristics on our experimental survey.

**Table 10:** Participants by segment memberships.

Segment count	N	Percent
0	1030	28%
1	1425	39%
2	838	23%
3	338	9%
4	37	1%

Total	3668	100%

*Notes:* We collected these demographic characteristics on our experimental survey.

**Table 11:** Effects of concordant message topics.

	Ordered logit	
	Intent to vaccinate self	Intent to vaccinate child
Concordant messages by topic		
Population tested	−0.013	−0.005
	[-0.228,0.201]	[-0.416,0.405]
Community impact	0.059	0.174
	[-0.073,0.191]	[-0.067,0.414]
Children affected by COVID-19	0.169	0.189
	[-0.059,0.397]	[-0.038,0.417]
Protecting the elderly	−0.026	0.018
	[-0.198,0.145]	[-0.223,0.259]
Protection from vaccine	−0.133	−0.095
	[-0.342,0.075]	[-0.446,0.256]
Gatherings made possible	0.062	0.058
	[-0.090,0.214]	[-0.156,0.272]
Vaccination locations	−0.026	−0.128
	[-0.223,0.172]	[-0.356,0.100]
Recommendation	0.020	−0.054
	[-0.147,0.186]	[-0.351,0.244]
ℙ(alltopics=0)	0.715	0.510
Subsegments	24	11
Observations	2621	1032

*Notes:* 95% confidence intervals in brackets using heteroskedasticity-robust standard errors. Regressions include subsegment fixed effects. Outcome ranges from 1 (highly unlikely) to 7 (highly likely). Concordant messages are the number of message components customized for that respondent’s segment memberships. Impact–Churches and Gatherings–Freedom were each a separate randomization, but analyzed as part of the Impact and Gatherings bundles, respectively. For example, everyone received one of the Impact messages, and half the Religious segment also received the Impact–Churches message.

^+^p<0.10, * p<0.05, ** p<0.01, *** p<0.001.

**Table 12:** Effects of concordant messages by segment

	Ordered logit	
	Intent to vaccinate self	Intent to vaccinate child
Concordant messages by segment		
Black	0.069	0.187
	[-0.109,0.247]	[-0.110,0.484]
Latinx	0.041	−0.059
	[-0.112,0.194]	[-0.385,0.267]
Conservative	0.007	0.061
	[-0.106,0.121]	[-0.128,0.249]
Religious	0.013	0.026
	[-0.113,0.139]	[-0.173,0.225]
Parent	−0.048	0.008
	[-0.165,0.070]	[-0.109,0.125]
ℙ(allsegments=0)	0.908	0.823
Subsegments	24	11
Observations	2621	1032

*Notes:* 95% confidence intervals in brackets using heteroskedasticity-robust standard errors. Regressions include subsegment fixed effects. Outcome ranges from 1 (highly unlikely) to 7 (highly likely). Concordant messages are the number of message components customized for that respondent’s segment memberships.

^+^p<0.10, * p<0.05, ** p<0.01, *** p<0.001.

**Table 13:** Effects of recommenders among conservatives

	Ordered logit (Reference recommender: Donald Trump)	
	Intent to vaccinate self	Intent to vaccinate child
Panel A. The effect of recommenders on intent to vaccinate		
The Obamas	−0.003	0.281
	[-0.392,-0.387]	[-0.423,0.985]
Dr. Fauci	−0.618**	−0.136
	[-1.012,-0.223]	[-0.847,0.576]
Dwayne “The Rock” Johnson	−0.305	−0.107
	[-0.695,-0.086]	[-0.890,0.676]
Tom Brady	−0.044	0.563
	[-0.449,-0.362]	[-0.161,1.288]
Tom Hanks	−0.332	0.241
	[-0.752,-0.089]	[-0.618,1.100]
The Pope^†^	−1.104*	
	[-1.969,-0.239]	
Rick Warren	−0.208	0.285
	[-0.786,-0.369]	[-0.577,1.147]
		
Panel B. Hypothesis tests of the effect of recommenders (p-values)		
All recs. = Trump	0.007**	0.347
The Obamas = Dr. Fauci	0.002**	0.161
The Obamas = The Rock	0.133	0.258
The Obamas = Tom Brady	0.844	0.351
The Obamas = Tom Hanks	0.133	0.919
The Obamas = The Pope	0.013*	
The Obamas = Rick Warren	0.476	0.991
Dr. Fauci = The Rock	0.123	0.933
Dr. Fauci = Tom Brady	0.006**	0.026*
Dr. Fauci = Tom Hanks	0.193	0.333
Dr. Fauci = The Pope	0.269	
Dr. Fauci = Rick Warren	0.160	0.277
The Rock = Tom Brady	0.212	0.060^+^
The Rock = Tom Hanks	0.900	0.414
The Rock = The Pope	0.073^+^	
The Rock = Rick Warren	0.744	0.353
Tom Brady = Tom Hanks	0.205	0.420
Tom Brady = The Pope	0.015*	
Tom Brady = Rick Warren	0.578	0.482
Tom Hanks = The Pope	0.091^+^	
Tom Hanks = Rick Warren	0.697	0.928
The Pope = Rick Warren	0.071^+^	

Recommender risk-sets^‡^	4	2
Observations	963	381

*Notes:* 95% confidence intervals in brackets using heteroskedasticity-robust standard errors. Outcome ranges from 1 (highly unlikely) to 7 (highly likely).

† Recommenders and recommender risk sets with fewer than three observations dropped.

‡ Regressions include recommender risk-set fixed effects.

^+^ p<0.10, * p<0.05, ** p<0.01, *** p<0.001

**Table:** 

Response	Participant count	Sample selection
United Kingdom	52285	
United States	44450	Required always
Ireland	1761	
Germany	2652	
…		

*Note:* Counts collected on 8 March 2021.

**Table:** 

Response	U.S. participant count	Sample selection
White	26181	
Black	3200	Selected
Asian	4403	
Mixed	2694	Selected
Other	1440	

*Note:* Counts collected on 10 May 2021.

**Table:** 

Response	U.S. participant count	Sample selection
African	155	
Black/African American	2557	
Caribbean	250	
East Asian	2587	
Latino/Hispanic	3180	Selected
Middle Eastern	314	
Mixed	2212	
Native American or Alaskan Native	235	
South Asian	964	
White/Caucasian	22587	
Other	260	
White/ Sephardic Jew	409	
Black/British	3	
White Mexican	113	Selected
Romani/Traveller	8	
South East Asian	666	

*Note:* Counts collected on 8 March 2021.

**Table:** 

Response	U.S. participant count	Sample selection
Joe Biden	12337	
Donald Trump	2400	Selected
Other candidate	837	
I did not vote	2648	
Rather not say or N/A	873	

*Note:* Counts collected on 8 March 2021.

**Table:** 

Response	U.S. participant count	Sample selection
Conservative	2131	Selected
Moderate	4274	
Liberal	8790	
Other	1153	
N/A	771	

*Note:* Counts collected on 10 May 2021.

**Table:** 

Response	U.S. participant count	Sample selection
Yes. Both public and private	5384	Selected
Yes. Public only	830	Selected
Yes. Private only	3184	Selected
None/ Rather not say	7206	

*Note:* Counts collected on 8 March 2021.

**Table:** 

Response	U.S. participant count	Sample selection
Yes (at least one dose)	762	
No	4349	Selected
Prefer not to answer	53	

*Note:* Counts collected on 8 March 2021.

## Appendix E Sample screening

Prolific participants may voluntarily answer a wide variety of survey questions written by Prolific. Researchers on the platform then may arbitrarily restrict their sample to participants who respond to any of these questions as desired. Note that not all participants answer each of the questions, so demographic characteristics inferred from these questions will be naturally underreported. Regardless, we use these response data to target the demographic segments of interest to our study.

Some Prolific participants took multiple screening surveys. For example, a Black conservative may have seen two (one because they matched on Black, one because they matched on conservative). These people will have multiple segment indicators values set. In this example, the respondent would have one screening indicator for Black and one for conservative.

Meanwhile, some Prolific participants took one screening survey, then when they opened a second, they realized it was identical and they returned the survey. This is because they wanted to avoid getting a duplicate survey rejected. So participants who have multiple screening indicator values may oversample dishonest people, forgetful people, and risk-tolerant people.

Note, however, that these values are only used for initial sampling. Our survey asks all respondents to report their relevant demographics. We use these responses to our own survey in our analysis.

**Country of residence**

We restrict our sample to individuals that currently reside in the United States.

*In what country do you currently reside?*

**Black or African American**

We use the following question to target individuals who identify as Black or African American.

*What ethnic group do you belong to?*

**Latinx or Hispanic**

We use the following Prolific question to target individuals who identify as Latina/o/x or Hispanic.

*Please indicate your ethnicity (i.e. peoples’ ethnicity describes their feeling of belonging and attachment to a distinct group of a larger population that shares their ancestry, colour, language or religion)?*

**Trump 2020 voters**

We target conservatives by selecting participants who reported voting for Trump in 2020.

*Who did you vote for in the 2020 US presidential election?*

**Politically conservative**

We also target conservatives who did not vote for Trump in 2020 using a political spectrum question.

*Where would you place yourself along the political spectrum?*

**Religious observation**

We target participants who participate in religious activities or observance.

*Do you participate in regular religious activities?*

**COVID-19 vaccine status**

Finally, we solicit some participants outside of our targeted demographic segments by seeking participants who had reported not having taken a COVID-19 vaccine dose.

*Have you received a coronavirus (COVID-19) vaccination?*

## Footnotes

1 The UCSB Human Subjects Committee exempted our Protocol 60-20-0658.

2 We pre-registered our study with the American Economic Association as AEARCTR-0007478 (Reddinger et al., 2021). We use Stata 17 and R 4.0.2 for analysis. Reddinger et al. (2022) provide data and source code.

3 Even an endorsement from Trump can be met with derision from conservatives; an audience booed Trump and Bill O’Reilly when in December 2021 they revealed having received a booster shot (Colvin, 2021).

4 Note that these authors only find significance at the 80% level with randomization-unit clustering.

5 Cairo et al. (2020) and Motyl et al. (2017) address publication bias in relevant literature.
